
JOURNAL INFORMATION
==============================
NLM Title Abbreviation: Tex Med J (Austin)
NLM Journal ID: 21130590R

Texas Medical Journal

ISSN: 0892-8495
Publisher: Biomedical Journal Digitization Project

ARTICLE INFORMATION
==============================
PMCID: PMC9591858
Article version: 1
Subjects: Books and Magazines

A Treatise on Diseases of the Skin

For the Use of Advanced Students and Practitioners

Print publication date: 1902 Sep
Volume: 18
Issue: 3
First page: 121
Last page: 122
License: This work is free of known copyright restrictions. For more information, please see PMC Back Issue Digitization.
License URL: https://creativecommons.org/publicdomain/mark/1.0/

==============================

Phillips' Emulsion 50% best NORWAY COD LIVER OIL minutely sub-divided, WITH WHEAT PHOSPHATES (Phillips')

Pancreatized, Palatable, Permanent, Miscible in Water, Milk, Wine, etc.

Phillips' Milk of Magnesia Mg H2 O2 (FLUID.) "THE PERFECT ANTACID."

for correcting Hyperacid conditions—local or systemic.
Vehicle for Salicylates, Iodides, Balsams, etc.

Phillips' Phospho-Muriate of Quinine, COMP.

TONIC AND RECONSTRUCTIVE.

WHEAT PHOSPHATES, WITH MURIATE OF QUININE AND STRYCHNINE.

PHILLIPS' WHEAT PHOSPHATES (ACID).
PHILLIPS' SYRUP OF WHEAT PHOSPHATES.
PHILLIPS' DIGESTIBLE COCOA.

THE CHAS. H. PHILLIPS CHEMICAL CO., NEW YORK

No physician can afford to be indifferent regarding the accurate filling of his prescription.

---

A Treatise on Diseases of the Skin. For the use of advanced Students and Practitioners. By Henry W. Stelwagon, M. D., Ph. D., Clinical Professor of Dermatology, Jefferson Medical College and Woman's Medical College, Philadelphia; Dermatologist to the Howard and Philadelphia Hospitals. Handsome octavo of 1125 pages, with 220 text-illustrations, and 26 full-page lithographic and half-tone plates. Philadelphia and London: W. B. Saunders & Co., 1902. Cloth, $6 net; Sheep or Half Morocco, $7 net.

In large treatises on dermatology an undue prominence is apt to be given the lengthy discussions of etiology and pathology, with the result that such works are often of but little value to any one except specialists. In this volume, fortunately, Dr. Stelwagon, bearing in mind, no doubt, the needs of the general practitioner, has had the good judgment to present in a full and comprehensive manner the essentially practical part of cutaneous medicine. He has offered every possible aid, both by text and illustration, to assist the student in the matter of diagnosis. Under "General Diagnosis" the signs and symptoms which serve to differentiate diseases that are apt to be confused, are carefully laid down and forcibly emphasized.

Though dealing chiefly with the essentially practical part of the diagnosis and treatment of skin diseases the work does not neglect etiology and pathology. In every instance the etiological factors are carefully outlined, and all of the established facts in pathology are clearly elucidated.

No just criticism of this volume can omit a special reference to its beautiful illustrations, most of which are from the author's own original collection. Both the text illustration in black and white and the colored lithographic plates are of exceptional merit.

---
